# Survival of immature *Anopheles arabiensis *(Diptera: Culicidae) in aquatic habitats in Mwea rice irrigation scheme, central Kenya

**DOI:** 10.1186/1475-2875-5-114

**Published:** 2006-11-24

**Authors:** Joseph M Mwangangi, Ephantus J Muturi, Josephat Shililu, Simon M Muriu, Benjamin Jacob, Ephantus W Kabiru, Charles M Mbogo, John Githure, Robert Novak

**Affiliations:** 1 International Centre for Insect Physiology and Ecology, Nairobi, Kenya; 2 Kenya Medical Research Institute, Centre for Geographic Medicine Research – Coast, P.O. Box 428, Kilifi 80108, Tel: +254- 41 522063; Fax +254- 41 522390, Kenya; 3 Center for Ecological Entomology, Illinois Natural History Survey, Champaign, Illinois 61820, USA; 4 Jomo Kenyatta University of Agriculture and Technology, Department of Zoology, Nairobi, Kenya; 5 Kenyatta University, Department of Pathology, Nairobi, Nairobi, Kenya

## Abstract

**Background:**

The survivorship and distribution of *Anopheles arabiensis *larvae and pupae was examined in a rice agro-ecosystem in Mwea Irrigation Scheme, central Kenya, from August 2005 to April 2006, prior to implementation of larval control programme.

**Methods:**

Horizontal life tables were constructed for immatures in semi-field condition. The time spent in the various immature stages was determined and survival established. Vertical life tables were obtained from five paddies sampled by standard dipping technique.

**Results:**

Pre-adult developmental time for *An. arabiensis *in the trays in the experimental set up in the screen house was 11.85 days from eclosion to emergence. The mean duration of each instar stage was estimated to be 1.40 days for first instars, 2.90 days for second instars, 1.85 days for third instars, 3.80 days for fourth instars and 1.90 days for pupae. A total of 590 individuals emerged into adults, giving an overall survivorship from L1 to adult emergence of 69.4%. A total of 4,956 *An. arabiensis *immatures were collected in 1,400 dips throughout the sampling period. Of these, 55.9% were collected during the tillering stage, 42.5% during the transplanting period and 1.6% during the land preparation stage. There was a significant difference in the *An. arabiensis *larval densities among the five stages. Also there was significant variation in immature stage composition for each day's collection in each paddy. These results indicate that the survival of the immatures was higher in some paddies than others. The mortality rate during the transplanting was 99.9% and at tillering was 96.6%, while the overall mortality was 98.3%.

**Conclusion:**

The survival of *An. arabiensis *immatures was better during the tillering stage of rice growth. Further the survival of immatures in rice fields is influenced by the rice agronomic activities including addition of nitrogenous fertilizers and pesticides. For effective integrated vector management, the application of larvicides should target *An. arabiensis *larvae at the tillering stage (early vegetative stage of rice) when their survival in the aquatic habitats is high to significantly reduce them and the larvicides should be long-lasting to have a significant impact on the malaria vector productivity on the habitats.

## Background

Vector-borne diseases are among the most important public health problems and obstacles to socioeconomic development of developing countries, particularly in the tropics, with malaria alone causing an estimated 1.5 – 2.7 million deaths and 300 – 500 million cases per year. Flood irrigation during rice cultivation has long been associated with an increase in number of disease vectors and corresponding increased health burden due to malaria and other vector and water-borne diseases. In the Mwea irrigation scheme, central Kenya, rice growing requires irrigation almost throughout the crop cycle. The *Anopheles gambiae *complex and the *Anopheles funestus *complex are the primary vectors of malaria in Mwea rice irrigation scheme. The distribution and abundance of mosquito larvae results from availability of oviposition sites, the oviposition preferences of females and the ability of the immatures to tolerate and develop after the eggs were laid.

Life tables provide a structured framework for identifying developmental stages most susceptible to mortality and, under some conditions, for inferring sources of larval deaths[1]. The life tables for the developing immatures can be constructed using either horizontal or vertical methods [1-4]. Horizontal life table methods are appropriate for distinct cohorts that can be followed through time, whereas vertical life table methods are appropriate for populations with overlapping generations and age distributions that remain stationary for the duration of the sampling period. Since vector abundance is a critical factor in the mosquito-malaria cycle, generational life-tables is a useful tool in malaria early warning systems for the purpose of forecast, early detection of epidemics and intensity of disease prevalence in holoendemic areas. Service [5-7], Reisen and Siddiqui [2] and Reisen et al.[4] provide extensive discussions about how such information can be analysed.

In Kenya, Service [5-7] and Aniedu [8] studied the survival of immature *An. gambiae *complex in the larval habitats. The objective of this study was to determine survival of immature *Anopheles arabiensis *in different habitats in the Mwea irrigation scheme and under the experimental rice growing conditions prior to implementation of a larval control programme.

## Methods

### Study site

The studies were conducted 100 km North East of Nairobi, in Mwea Irrigation and Agricultural Development Center (MIAD) within the Mwea irrigation scheme. Mwea occupies the lower altitude zone of Kirinyaga District in an expansive low-lying area. The mean annual rainfall is 950 mm with maximum amount falling in April/May (long rains) and October/November (Short rains). The average maximum temperatures are in the range of 16–26.5°C. Relative humidity varies from 52–67%. According to the 1999 national census, the Mwea irrigation scheme has approximately 150,000 persons in 25,000 households. The Mwea rice irrigation scheme is located in the west central region of Mwea Division and covers an area of about 13,640 ha. More than 50% of the scheme area is used for rice cultivation. The remaining area is used for subsistence farming, grazing and community activities.

Adult mosquito sampling was done at the Kariwa village, which is nearby the MIAD center. Kariwa has approximately 68 homesteads with over 350 residents. Cows, goats, chickens, and donkeys are the primary domestic animals. These animals are kept within 5 m of most houses. Over 95% of the houses have mud walls with iron roofing.

### Horizontal life tables semi field condition

Blood-fed *An. arabiensis *females were collected with aspirators from houses [9] throughout Kariwa village and kept in cages containing 2% sugar solution in a semi field condition at the Mwea Irrigation and Agricultural Development Center (MIAD) field station until they became gravid. A screen house constructed at the MIAD centre provided the semi field condition. Petri dishes containing a small amount of distilled water were placed inside the cages to attract oviposition. Newly laid eggs in these containers were monitored every 0.5 h to determine their time of eclosion. Approximately 2 h after eclosion, 50 first-instar larvae (L1) were collected for each replicate. These were transferred to larval rearing pans (30 by 24 cm) at an initial density of 14 larvae/cm^2 ^of pan surface area (L/cm^2^). The screen house provided a semi field condition to the mosquito larvae during the entire development period. Briefly the screen house was constructed with wooden framework and plastic mosquito netting pinned at the sides. The plastic netting material was reinforced with chicken wire mesh. The roof was made of translucent roofing material ensuring the penetration of sunlight. The mosquito proof netting ensured that any ovipositing adults were excluded from the trays inside the screen house. The mosquito larvae rearing were done using water collected from the inlet canal to the paddies in the field station. The nutrient for larvae development was found in the floating particles in this water. The water was changed in the trays every other day. Room temperature and relative humidity were monitored daily every three hours using a HOBO^® ^(Onset Computer Corporation, Bourne, Massachusetts, USA). BoxCar Pro (Onset Computer Corporation, Bourne, Massachusetts, USA) was used to download the weather information at the end of the study duration.

Duration of the preadult development period were determined by observing each pan at 09.00 and 16.00 h daily, at each time all larval skins were removed, scored to instar, and counted. The larval cuticle of L1 was identified by distinctive dark head capsules with semi-transparent abdominal exuviae at around 24 to 40 h after the pans were established. The number of larvae metamorphosing to the next instar could thus be inferred. The time taken for 50% of the larvae in a pan to change to the next instar was taken as the median developmental period of that specific stage. Computations of immature stage duration and survivorship followed those of Reisen et al.[4]. A sub sample of emergent *An. gambiae s.l*. adults were further identified using rDNA Polymerase Chain Reaction (PCR) technique into the sibling species [10].

When all adults had emerged, stage-specific survivorship for the pooled data was estimated according to method used by Edillo et al [11]:

***S*i **= ***n*_i_**/**(*n*i - 1),**

where

*n*i = total number of immatures entering life instar *i*,

and *n*i - 1 = the number alive in the previous instar.

Mean instar duration in hours at molting, *T*i, was:

***D*i **= ***T*i **- **(*t*_i-1_),**

where *t*_i-1 _was the previous mean age at molting.

The percentage of total immature life spent at each instar was:

***L*_i _**= **100 × *D*i**/***t*_5_,**

where *t*_5 _was the median time of adult emergence.

Relativized probability of capture in a vertical sample was ***P*_i _**= ***D*_i_**/***D*_p_,**

where *D*_p _was the duration of the shortest-lived life stage, which was taken as the standard.

Survivorship from L1 to adult emergence was estimated by ***A/I***,

where *A *= total number of adults

and *I *= total number of L1 originally counted into the rearing trays.

### Vertical life table

Five paddy habitats were randomly selected in MIAD centre rice fields for the generation of vertical life tables. Larval sampling was done using standard dipping technique. The larvae collected per dip were counted and scored to life stage [12]. The contents of each dipper were poured into a white tray and the mosquito larvae counted and age-graded. The larvae were grouped according to species and age graded as early instars (L1, L2), late instars (L3, L4) and Pupae (P). The larval sampling was done in from land preparation to rice tillering stage. This period of rice growing has been associated with increase in *Anopheles *larval densities and diversity [13]. In each season the larval habitats selected was sampled four times (once every week). A sub-sample of *An. gambiae s.l*. late stage instars larvae were further identified using rDNA Polymerase Chain Reaction (PCR) technique into the sibling species [10].

### Numbers of immatures in each habitat

The distribution of immatures per habitat was compared in a contingency table. The immature age *An. arabiensis *was categorized as early instars (L1 + L2), late stage instars (L3 + L4) and pupae (P), and the chronological collection periods were based on rice growth cycle grouped as land preparation, transplanting and tillering stage. These were then analysed statistically as a 3 (age groups) × n (habitats) × n (sampling periods) contingency table.

### Measurement of physicochemical variables

Temperature, pH, conductivity, dissolved oxygen, salinity, Total Dissolved Solids (TDS), nitrates, phosphates, ammonia and sulphate content were measured in the paddies. Briefly, pH, conductivity, dissolved oxygen and temperature was measured using hand held machine YSI 650 Multiparameter Display System (YSI Environmental, YSI Incorporated, Yellow Springs, USA). Salinity and Total Dissolved solids (TDS) were measured using field hand held equipment YSI EC 300 (YSI Environmental, YSI Incorporated, Yellow Springs, USA). Nitrates, phosphates, ammonia and sulphate content were measured using spectrophotometric technique by use of portable HACH machines (HACH^®^, DR/2400 Spectrophotometer, Hach Company, Ames, Iowa). All physicochemical parameters were measured on site at the time of mosquito larval sampling.

## Results

### Age distribution and survivorship curves

Pre-adult developmental time in the trays in the experimental set up in the screen house was 11.85 days (d) (284.40 h) from eclosion to emergence (Table 1). The mean duration of each instar stage *i*, D_*i*_, were estimated to be 1.40 days (d) (33.60 h) for first instars, 2.90 d (69.60 h) for second instars, 1.85 d (44.40 h) for third instars, 3.80 d (91.20 h) for fourth instars and 1.90 d (45.60 h) for pupae. The survivorship rate was estimated to be 92.94% during L1 stage, 98.1% for the L2 stage, 93.55% for the L3 stage, 83.44% for the L4 stage, and 97.52% for pupae. A total of 590 individuals emerged into adults, giving an overall survivorship from L1 to adult emergence of 69.41%. A sub-sample of 258 emergent *An. gambiae s.l*. was further analysed using rDNA PCR technique and confirmed *An. arabiensis *as the only sibling species present. The emergent *An. arabiensis *mosquitoes were composed of 354 females (60%) and 236 males (40%). It was observed that the males emerged first, then followed by the females and the mosquitoes emerged in the evening.

**Table 1:** Instar mortalities of *Anopheles arabiensis *at the screen house

Instars **(i)**	Age at the beginning of Instar **(ti-1)**	No. entering instar **(Sti-1)**	Deaths in instar **(Di)**	Relative proportion dying in Instar **(Di/Sti-1)**	Proportion dying daily in instars **1-(Sti/Sti-1)^1/d^**
L1	0	850	60	0.071	0.051
L2	1.40	790	15	0.019	0.007
L3	4.30	775	50	0.065	0.035
L4	6.15	725	120	0.166	0.047
Pupa	9.95	605	15	0.025	0.013
Adult	11.85	590			

**Figure 1:**
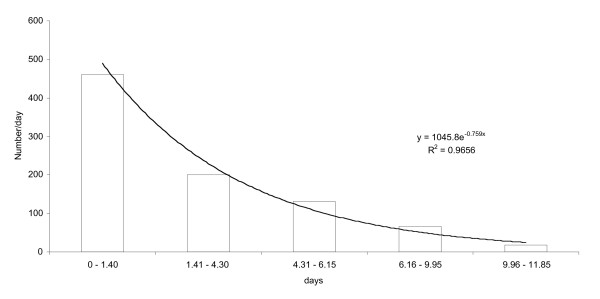
Age distribution and survivorship curve for the immature stages (1^st ^instar – Pupae) of *Anopheles arabiensis*.

The larvae per instars stage were plotted into a histogram and this resulted into the age distribution histograms, which was then fitted with a best-fit curve to give the age specific curve, which simulates the time specific survivorship curves (Figure 1). Overall survivorship curves for immature stages of *An. arabiensis *in trays in screen house were pooled due to the general similarity in the two-week period survivorship curves. Using the outline of Southwood [14] life tables for *An. arabiensis *immature stages were calculated from these survivorship curves as shown in Figure 1.

### Vertical life tables

A total of 4,956 *An. arabiensis *immatures were collected in 1,400 dips throughout the sampling period (Table 2). Of these, 2,771 (55.91%) were collected during the tillering stage, 2,105 (42.47%) transplanting period and 80 (1.61%) during the land preparation stage. Further analysis using rDNA PCR technique confirmed that *An. arabiensis *was the only sibling species present in Mwea Irrigation Scheme. There was significant variation in immature stage composition for each day's collection in each paddy (F_(2,64) _= 3.829, p = 0.007). Most late stage and pupae of *An. arabiensis *were collected at the tillering stage. The lowest densities of late stage and pupae were collected at the land preparation stage. If the distribution of the immature stages in the paddies was random and uniform, the distribution should be Poisson, with the variance/mean equal to 1. The coefficient of dispersion in this study were consistently was >1, implying aggregation or clumping of immatures (Table 3). Two paddies (C and D) had a large coefficient of dispersion indicating that the *An. arabiensis *larvae were highly aggregated in some parts of the paddy. The most common families of other non-mosquito invertebrates observed in the paddies were Dytiscidae, Hydrophillidae, Notonectidae, Ephemerellidae, Coenagrionidae, Lebullidae and Belostomatidae.

**Table 2:** The total number of *An. arabiensis *immatures collected from paddies at each rice growth cycle

**Rice stage**	**Early instars *Anopheles***	**Late instars *Anopheles***	**Pupae**
Land preparation	50	23	7
Transplanting	1,743	300	62
Tillering	2,045	460	266

**Total**	**3,838**	**783**	**335**

**Table 3:** The Mean, variance (s^2^) and coefficient of dispersion (CD) of the densities of *An. arabiensis *larvae collected from each paddy.

**Paddy number**		**Early instars**	**Late instars**	**Pupae**
A	Mean	1.18	0.34	0.05
	Variance	2.57	0.44	0.07
	CD	2.18	1.27	1.46

B	Mean	0.94	0.23	0.01
	Variance	1.00	0.96	0.03
	CD	1.07	4.12	4.62

C	Mean	6.20	0.96	0.75
	Variance	78.34	1.76	2.50
	CD	12.63	1.83	3.32

D	Mean	3.90	1.02	0.25
	Variance	34.32	1.85	1.31
	CD	8.81	1.81	5.30

F	Mean	1.33	0.17	0.11
	Variance	4.76	0.75	0.15
	CD	3.57	4.48	1.30

Total	Mean	2.71	0.55	0.23
	Variance	27.06	0.93	0.60
	CD	9.99	1.71	2.56

### Physicochemical conditions of the paddies

Table 4 shows the mean values of the physicochemical parameters in paddies during each rice growing cycle. The micronutrients measured, nitrate, sulphate and nitrogen ammonia were low at land preparation but increased at transplanting and tillering due to the addition of basal and top dressing fertilizers. Temperature was lowest at land preparation but increased slightly after transplanting. pH remained stable during the sampling period. Salinity and conductivity was highest at the land preparation but declined after transplanting. There was, however, no significant difference in the physicochemical variables composition between transplanting and tillering (p > 0.05).

**Table 4:** Mean and Standard Error (SE) of physicochemical conditions of water samples taken from paddies with different rice stage

**Rice stage**	**Land preparation**	**Transplanting**	**Tillering**
Temperature	22.8 ± 0.87	24.25 ± 1.07	24.22 ± 0.70
Conductivity (ms/cm)	301.5 ± 45.11	317.20 ± 60.30	178.72 ± 15.45
Salinity (ppt^a^)	319.54 ± 23.01	240.44 ± 45.07	159.52 ± 18.32
DO^b ^(%)	0.2 ± 0.01	64.92 ± 14.04	91.89 ± 13.71
pH	7.86 ± 0.15	7.85 ± 0.10	7.61 ± 0.06
Nitrate (mg/l)	13.6 ± 3.44	25.9 ± 4.99	12.64 ± 5.09
Sulphate (mg/l)	80 ± 5.25	54.4 ± 11.00	68.22 ± 28.59
Nitrogen Ammonia (mg/l)	0.01 ± 0.001	4.1 ± 0.080	0.14 ± 0.02

Legend: ppt^a ^Parts Per Thousand, DO^b ^Dissolved Oxygen

### Distribution of immatures *Anopheles arabiensis *in the paddy

**Figure 2:**
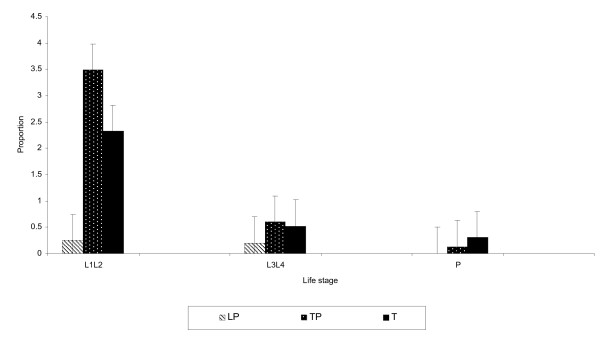
**The proportion of *Anopheles arabiensis *immatures at the paddy for each rice growth stage sampled at the MIAD center at the Mwea Irrigation Scheme**. Legend: **LP **Land preparation, **TP **Transplanting, **T **Tillering.

The distribution of immatures in the age classes for each age class and rice growth cycle is shown in Figure 2. There was a significant difference in the *An. arabiensis *larval densities among the five paddies (F_(2,12) _= 4.94, p = 0.027). These results further indicate that the survival of the immatures was higher in some paddies than others. The mortality rate during the transplanting was 99.95% and at tillering was 96.61% and the overall mortality was 98.26%. The survival of the immatures was best at tillering and more adult production (as indicated by number of pupae) was highest at this rice growth stage.

## Discussion

This study found that survivorship of *An. arabiensis *larvae in paddies was higher at the tillering stage than at the transplanting stage. At the tillering stage there is addition of inorganic nitrogenous fertilizers. The addition of the nitrogenous fertilizers acts as the attractant for oviposition by the gravid *An. arabiensis *mosquitoes. Broadcasting nitrogenous fertilizers in rice fields has been found to enhance mosquito larval populations [15,16]. Also few days after transplanting, there is addition of nitrogenous fertilizers, which has been shown to attract *Anopheles *and culicine mosquito oviposition [16,17]. Furthermore, there was a strong positive correlation in between fertilizer application and physicochemical variables tested. There was an increase of nitrates, sulphate, nitrogen ammonia and dissolved oxygen immediately after transplanting which is attributed to addition of nitrogen fertilizers. It was hypothesized that the changes in these physicochemical variables acted first as oviposition attractant resulting in higher densities of *Anopheles *larvae and secondly high nutritional resources, which ensured high survivorship of these larvae in the paddies. The nitrogenous fertilizer application enhanced the food resource available for the anopheline immatures stages that increases their survival and eventually resulting to higher vector productivity.

The study has demonstrated that survivorship of *An. arabiensis *mosquito larvae from first instar to adult emergence was lower in the paddies. This is an indication that a higher proportion of larvae and pupae died in the paddy habitats. The findings in this study were similar to that of Sunahara et al. [18] who found out that the causes of mortality in the rice fields were caused by both abiotic and biotic factors. Service [7] attributed the efficacy of the natural enemies in such small habitats that may be inhabited by mosquito larvae to the small volume of water in them. When eggs are laid in a given habitat, mosquito competitors and predators can reduce mosquito densities by direct mortality and/or reduced growth rates due to reduced activity and due to reduced food availability.

In the experimental set up, there was significant reduction in the immatures. In the trays all larval instars were found together. Koenraadt and Takken [19] showed that within the habitats, all four larval instars were found together. In this set up majority of dead larvae were not recovered from the trays with L4 present suggesting that there was cannibalism occurring at the trays. Cannibalism and predation are important factors in reducing the larval survivorship in the habitats especially the small sized habitats. Koenraadt and Takken [19] and Koenraadt [20] showed that the older larval instars (L4) in the trays reduced the numbers of earlier instars stages.

This study also demonstrated that high proportion of larvae died in the 1^st ^and 3^rd ^instars as well as in pupal stage. The overall mortality was significantly high in all the habitats, being 98.26%. These high mortality rates are consistent with survivorship rates results of *An. arabiensis *and with larval mortality rates results by other workers under field conditions. Service [7] recorded 92.6–100% mortality for *An. gambiae *rice fields and temporary pools, Kano plains Kisumu, while Aniedu et al. [8] recorded a 91.9% for *An. gambiae *in Baringo, Kenya. The high mortality in the present study can be attributed partly to predation since presence of known and potential *An. arabiensis *larval and pupal predators were recorded although quantification of the relative contribution or proportion of mortality of the mosquito immature stages that was due to predation was not conducted. Some of the families of the other non-mosquitoes invertebrates observed in this study have been noted as potential predators in some previous studies in western Kenya [6,7]. However, previous estimates indicate that significant mosquito larval and pupal population reduction, 50–90% in *Culex tritaeniorhynchus *[2] and 82% in *An. gambiae *mosquitoes [21] was due to predation effects.

It was further observed that when the paddies were sprayed with Fenitrithion was applied at a rate of 400 ml/acre, 35 days after transplanting to control insect pests, all the *An. gambiae *larvae died and the survival was zero. The Fenitrithion also killed all the other invertebrates within the paddy indiscriminately. The zero percent mortality continued for about ten days when the colonization started again in the paddies. The application of the pesticide was done together with topdressing with sulfate of ammonia. The nitrogenous fertilizer attracted the gravid *Anopheles *mosquitoes were at the same time attracted to oviposit consequently with the diminishing effect of the pesticide the *An. arabiensis *larval densities and survival increased significantly. This increased the survivorship of immatures significantly as the other non-mosquito invertebrates were very low in densities. More detailed ecological studies are required to elucidate the importance of predators and pathogens in regulating population size of the immature stages of *An. arabiensis *in the rice fields of Mwea Irrigation Scheme during the rice growing cycle and to identify other limiting factors governing their numbers and local distribution. In conclusion, the survival of *An. arabiensis *immatures was better during the tillering stage of rice growth. Further the survival of immatures in rice fields is influenced by the rice agronomic activities including addition of nitrogenous fertilizers and pesticides. For effective integrated vector management, the application of larvicides should target *An. arabiensis *larvae based on the early developmental stages of rice. The larvae should be targeted at the tillering stage (early vegetative stage) when their survival in the aquatic habitats is high to significantly reduce them and the larvicides should be long lasting to have a significant impact on the malaria vector productivity on the habitats.

## Competing interests

The author(s) declare that they have no competing interests.

## Authors' contributions

JMM, EJM and SMM conducted all the experimental work. JS provided scientific guidance in data collection, analysis and manuscript preparation and planning, and implementation of day-to-day field activities. BJ, CMM and EWK, offered scientific guidance in data analysis and manuscript preparation. JG and RN provided overall supervision of the study and preparation of manuscript. All authors actively contributed to the interpretation of the findings and development of the final manuscript and approved the final manuscript.
